# Association of Endothelin-1 Expression and Cartilaginous Endplate Degeneration in Humans

**DOI:** 10.1371/journal.pone.0060062

**Published:** 2013-04-02

**Authors:** Wei Yuan, Ming-Dong Zhao, Feng-Lai Yuan, Wu Che, Ping-Guo Duan, Yi Liu, Jian Dong

**Affiliations:** 1 Department of Orthopedic Surgery, Zhongshan Hospital, Fudan University, Shanghai, China; 2 Department of Orthopedic Surgery, Jinshan Hospital, Fudan University, Shanghai, China; 3 Third Hospital of Nantong University, Wuxi, China; 4 Department of Orthopedic Surgery, The First Affiliated Hospital of Nanchang University, Nanchang, China; 5 Department of Orthopedic Surgery, Huadong Hospital, Fudan University, Shanghai, China; University of Medicine and Dentistry of New Jersey, United States of America

## Abstract

**Background:**

Inflammatory cytokines are involved in intervertebral disc (IVD) degeneration. Endothelin-1 (ET-1), a 21-amino-acid cytokine implicated with cartilage degradation, is secreted by vascular endothelial cells and also by many other cell types. The expression of ET-1 in human IVD cartilage endplate (CEP) and its role in disc degeneration have not been explored.

**Methods and Findings:**

The expression of ET-1 in degenerated CEP was analyzed by immunohistochemical staining and Western blotting; ET-1 was demonstrated in cartilaginous endplate cells (CECs) by immunofluorescent staining. The ET-1 mRNA expression and protein production by CECs stimulated by tumor necrosis factor alpha (TNF-α), a pro-inflammatory cytokine, were determined by real-time PCR analysis and Western blotting, respectively. The matrix metalloprotease-1 (MMP-1), MMP-13 and tissue inhibitor of metalloproteases-1 (TIMP-1) levels in the supernatant of cultured CECs treated with ET-1 were determined using enzyme-linked immunosorbent assays. Nitric oxide (NO) release and nitric oxide synthase (NOS) activity were measured using a spectrophotometric assay. The apoptosis of CECs by ET-1 was measured by an Annexin V-FITC detection assay. The production of ET-1 in degenerated cartilage endplate was significantly higher than normal CEP. The results showed that ET-1 was expressed by CECs and modulated by TNF-α in a dose-dependent manner. ET-1 increased production of MMP-1 and MMP-13, decreased TIMP-1 production, and induced NO and NOS release by cultured CECs. The direct stimulation of CECs by ET-1 did not promote cell apoptosis.

**Conclusion:**

The study results suggest that ET-1 played a pivotal role in human CEP degeneration, and may be a new target for development of therapies for this condition.

## Introduction

Vertebral endplates form the superior and inferior boundaries of the vertebral bodies, which articulate with intervertebral discs (IVD). They are composed of a thin layer of cortical bone covered by hyaline cartilage produced by chondrocytes. The IVD depends for its nutrition on the vascular supply from surrounding tissues including the vertebral body [1], [2].

Cartilage endplate (CEP) degeneration is characterized by matrix disorganization and loss of substance, which can be caused by proteinase activity. The morphological changes that characterize endplate degeneration include subchondral sclerosis, calcification of the hyaline cartilage and fissure formation, resulting in structural disorganization. On the molecular level, the metabolism and biosynthetic functions of the cartilaginous endplate cells (CECs) decrease as the matrix becomes degraded [3]. The activity of matrix metalloproteinases (MMPs) is high in degenerative discs, and the balance between production of tissue inhibitors of metalloproteinase (TIMP) and MMPs appears to be altered [4]. This is accompanied by the induction of collagenases (MMP-1 and MMP-13) that are known to be involved in disc degeneration. MMP-1 (collagenase 1, interstitial collagenase) and MMP-13 (collagenase 3) are of particular importance, as they can cleave intact triple-helical collagen molecules [5]. MMP-13 preferentially cleaves type II collagen [6]. Anderson *et al.* confirmed that degenerative disc changes are associated with up-regulation of collagenases MMP-1 and MMP-13 [7]. In addition, human herniated lumbar disc cultures spontaneously produce nitric oxide (NO), a known mediator of proteoglycan synthesis [8], [9].

It has been reported that inflammatory cytokines are involved in the pathogenesis of IVD degeneration [10]. Endothelin-1 (ET-1) has been recognized as one of the most potent vasoconstrictor agents [11]. ET-1 was firstly discovered in aortic endothelial cells, and has since been found to be produced by many cell types [12]. Interestingly, ET-1 is not only a potent vasoconstrictor, but is also associated with inflammation in degenerative diseases mainly via endothelin receptor type A. ET-1 causes excessive production of NO, which is generated following increases in inducible nitric oxide synthase (NOS) levels [13]–[15]. In addition, ET-1 promotes MMP-1 and MMP-13 synthesis and activation in osteoarthritis cartilage [16].

As mentioned above, recent research has shown that ET-1 is an inflammatory cytokine involved in cartilage degenerative disease. It is not known if ET-1 is expressed by chondrocytes in human IVD endplates or if it mediates pathologic processes there. Therefore, the aim of this study was to determine if ET-1 is produced in human CEP and if activation or over-expression of ET-1 could alter the synthesis and retention of cartilage matrix molecules, MMPs, or otherwise play an important role in IVD tissue degeneration.

## Materials and Methods

### Ethics Statement

The Institutional Ethics Committee Board of Zhongshan Hospital, Fudan University approved the study protocol and the use of human tissues. All study participants gave written informed consent before enrolment.

### Study Design

The CEPs used in this study was obtained from eight patients with lumbar degenerative disease. Control tissue specimens were obtained from eight patients, seven with acute burst fractures of lumbar vertebra and one with lumbar neural arch cysts but no signals of disc degenerative or Modic changes in CEPs on MRI. All participants underwent posterior discectomy and fusion for lumbar disease. The status of lumbar disc degeneration was described according to the modified Pfirrman grading system [17]. Descriptions of all specimens are shown in Table 1.

**Table 1:** Study-related patients information.

Case no.	Diagnosis	Disc level	Modified Pfirrman grade	Gender	Age
1	Lumbar disc herniation	L3–L4	3	M	44
2	Spondylolisthesis	L4–L5	4	M	42
3	Spinal stenosis	L5–S1	4	M	55
4	Lumbar disc herniation	L4–L5	5	F	65
5	Spondylolisthesis	L4–L5	4	F	59
6	Lumbar disc herniation	L5–S1	5	M	40
7	Spondylolisthesis	L4–L5	3	F	48
8	Lumbar disc herniation	L4–L5	4	M	50
9	Vertebral fracture	L3	1	M	28
10	lumbar neural arch cysts	L4	1	M	41
11	Vertebral fracture	L5	1	F	32
12	Vertebral fracture	L2, L4	1	M	35
13	Vertebral fracture	L4–L5	1	F	30
14	Vertebral fracture	L1–L2	1	M	22
15	Vertebral fracture	L2	1	M	29
16	Vertebral fracture	L1	1	M	25

### Isolation and Culture of Cartilaginous Endplate Cells (CECs)

The surgically harvested endplate cartilages were carefully cleaned of nucleus pulposus and annulus fibrosus. After washing with phosphate buffered saline (PBS), a small piece of each endplate was prepared for hematoxylin and eosin (H&E) and immunohistochemical (IHC) staining. A second sample (about 70 mg) was reserved for western blot assay. The remainder of each sample was minced into pieces <1 mm^3^ with sterile ophthalmic scissors, and digested with 0.15% collagenase type II (Invitrogen, USA) in Dulbecco’s Minimum Essential Medium (DMEM) (Invitrogen, USA) containing 5% fetal calf serum (FCS) for 12 h at 37°C with shaker agitation. The cell suspension was passed through a 70 µm filter to remove aggregates and was then centrifuged for 10 min at 2000 rpm. The supernatant was discarded; the cells were washed three times with PBS and centrifuged again to obtain a pellet. Finally, the cells were cultured in 8 cm^2^ Petri dishes (Costar Corning, USA) in DMEM with 10% FCS and 1% penicillin/streptomycin. Cultures were incubated at 37°C and 95% relative humidity in a 5% CO_2_ atmosphere. Cells for all experiments were used at the third passage of culture after isolation.

### Histochemical Staining

The morphology of cultured cells was evaluated in H&E. To confirm the deposition of sulfated glycosaminoglycan, cell preparations were incubated with 0.1% toluidine blue (0.1 g toluidine blue+2 g NaHCO_3_+100 ml distilled H_2_O) at 37°C for 30 min. Cultures were washed extensively with distilled water and photographed.

### Immunohistochemical Staining

IHC staining for ET-1 was performed in IVD samples. Briefly, 4 µm sections were deparaffinized and incubated with 3% hydrogen peroxide for 15 min. After blocking endogenous peroxidase activity, antigen retrieval was performed by heating the sections in citrate buffer. After washing three times in PBS containing 0.1% Triton X, the samples were blocked with a solution containing 5% rabbit serum albumin and 0.1% Triton X for 1 h. Then, the primary antibody of goat anti-human ET-1 (Santa Cruz, CA, USA) was diluted 1∶50 and applied overnight at 4°C. Negative controls were incubated without the primary antibody. Corresponding secondary anti-goat IgG antibody labeled with horseradish peroxidase (HRP) (CW-BIO, Beijing, China) was applied at 1∶200 dilution for 1 h at room temperature. Positive staining was shown by the presence of brown 3, 3′-diaminobenzidine (DAB) color. Nuclei were counterstained blue with hematoxylin. The stained preparations were photographed with an Olympus BX51 microscope (Olympus, Tokyo, Japan).

CECs were also assayed by IHC staining for collagen II and aggrecan. Cells were seeded on glass slides in 12-well plates at a density of 50,000 cells per well. All assays were performed in triplicate wells. After attachment, the cells were fixed in 4% paraformaldehyde, incubated for 20 min with 3% hydrogen peroxide, blocked with 1% bovine serum and incubated with rabbit anti-human type II collagen (1∶200, Abcam, Cambridge, MA, USA) and rabbit anti-human aggrecan (1∶200, Abcam, Cambridge, MA, USA) monoclonal antibody overnight at 4°C in a humidified chamber. After washing with PBS, the cells were incubated with an HRP labeled anti-rabbit IgG antibody (CW-BIO, Beijing, China) at room temperature for 1 h. Positive staining was revealed by formation of 3, 3′-DAB. The samples were counterstained with hematoxylin.

### Immunofluorescence

CECs were seeded in 12-well plates; all assays were performed in triplicate. When confluence was reached, the cells were fixed in acetone and treated with a blocking solution containing 5% rabbit serum and 0.1% Triton X. The cells were stained with primary polyclonal goat anti-human ET-1 antibody (1∶50, Santa Cruz, CA, USA) overnight at 4°C. They were then washed three times in PBS and stained for 1 h with fluorescein isothiocyanate (FITC)-conjugated rabbit anti-goat IgG secondary antibody (1∶100, CW-BIO, Beijing, China). Cells were then treated with the fluorescent stain, 2-(4-amidinophenyl)-6-indolecarbamidine dihydrochloride (DAPI; 2 µg/ml, Beyotime, Zhengzhou, China). Negative controls were obtained by omission of the primary antibody and by incubation with a blocking solution. Positive cytoplasmic staining was observed with a fluorescence microscope.

### Quantitative Real-time Polymerase Chain Reaction (PCR) for ET-1

Pro-inflammatory cytokines such as TNF-α can stimulate expression of many inflammatory factors. Real-time PCR was performed to confirm the expression of the ET-1 mRNA in CECs. Cultured cells were seeded into 6-well plates at a density of 3×10^5^ cells per well. All assays were performed in triplicate. After attachment, cells were incubated with fresh serum-free medium for 12 h and were then treated with TNF-α (0–100 ng/ml) in fresh serum-free DMEM for 24 h. For real-time PCR, 1 µg of RNA and reverse-transcribed cDNA were used. SYBR Green dye (Takara, Dalian, China-Japan) was used to detect DNA synthesis. The following ET-1 primers were used: 5′-GACATCATTTGGGTCAACACTC-3′ for the forward primer and 5′-GGCATCTATTTTCACGGTCTGT-3′ for the reverse primer. The following glyceraldehyde-3-phosphatedehydrogenase (GAPDH) primers were used: 5′-AGAAGGCTGGGGCTCATTTG-3′ for the forward primer and 5′-AGGGGCCATCCACAGTCTTC-3′ for the reverse primer. The mRNA levels were normalized against the housekeeping gene GAPDH. The real-time PCR was applied in the Rotor Gene real-time DNA amplification system using the following cycling protocol: a 95°C denaturation step for 30 s, followed by 40 cycles of 95°C denaturation (5 s), 60°C annealing (20 s), and 72°C extension (20 s). PCR products were subjected to melting curve analysis, and the data were analyzed and quantified using Rotor Gene analysis software.

### Western Blotting for ET-1 Production

CEPs were surgically obtained from four donors at random (two normal CEPs and two degenerated CEP samples) for protein analysis. The proteins were resolved by SDS-PAGE in a 12% polyacrylamide gel and transferred to a polyvinylidene fluoride membrane by electroblotting. The membranes were blocked in tris-buffered saline supplemented with Tween and 5% nonfat dry milk for 2 h at room temperature with constant agitation. After incubation with primary antibody of goat anti-human ET-1 (1∶200, Santa Cruz, CA, USA) overnight at 4°C, the secondary rabbit anti-goat HRP-conjugated antibody (CW-BIO, Beijing, China) was added and incubated for 1 h. Immunoreactive proteins were assayed by chemiluminescence (ECL, Beyotime, Zhengzhou, China) and analyzed by Image J 1.43 software.

Western blotting was used to correlate increase in ET-1 levels in cultured cells following TNF-α treatment. Cultured cells were plated in 6-well plates in DMEM, supplemented with 10% FCS and 1% penicillin-streptomycin. Conditioned media were assayed in duplicate. When the cells were approximately 85–90% confluent, the culture medium was replaced with serum-free medium for 12 h for synchronization, and then with serum-free medium containing various concentrations of TNF-α (0–100 ng/ml) for 36 h. The cells were lysed, and the protein concentrations were quantified. Subsequent steps were carried out following the same procedure described above.

### Enzyme-linked Immunosorbent Assay (ELISA)

To evaluate the effects ET-1 (Sigma, Louis, MO, USA) on CECs release of MMP-1, MMP-13 and TIMP-1, ELISA was performed in 24-well plates after ET-1 (0–100 nM) treatment for 36 h. Prior to treatment, confluent cells were incubated with serum-free DMEM for 12 h. All assays were carried out in triplicate. Following incubation, the MMP-1 MMP-13 (RayBiotech, Norcross, GA, USA) and TIMP-1 (R&D, Minneapolis, MN, USA) protein levels in the supernatants were assayed using a specific ELISA kit according to the manufacture’s protocol. The absorbance was read at 450 nm with a microplate reader; results were analyzed by comparing absorbance values against a standard curve.

### Quantization of NO Content and NOS Activity

NO and NOS were assayed spectrophotometrically (NJJC-BIO, Nanjing, China) at absorbances of 550 nm and 530 nm, respectively, in CECs culture supernatants following treatment with ET-1 (0–100 nM). All assays were performed in triplicate.

### Apoptosis

Apoptosis of cultured CECs was measured following ET-1 treatment. At confluence, the cells were incubated at 37°C for 72 h in DMEM containing 2% FCS in the presence of ET-1 (0–100 nM). Apoptotic cells were detected with an Annexin V-FITC/PI Apoptosis detection kit (KeyGEN-BIO, Nanjing, China), the results were obtained by flow cytometric analysis.

### Statistical Analysis

The data were expressed as mean ± SD of three independent experiments. The results were analyzed via one-way analysis of variance (ANOVA) to compare differences between variables in different groups, and *P*<0.05 was considered significant.

## Results

### Expression of ET-1 in Human Degenerated CEPs

**Figure 1:**
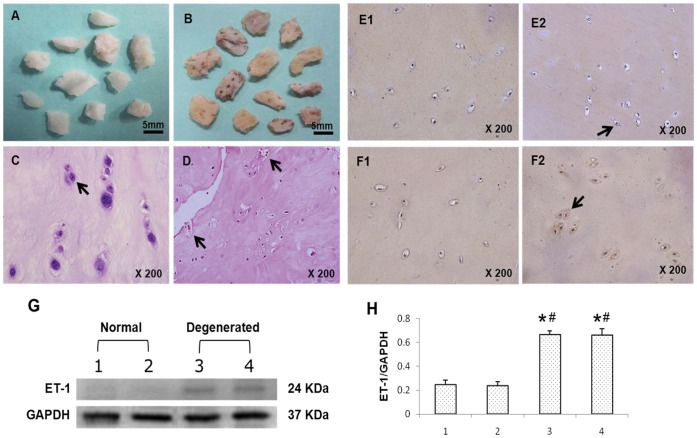
Morphological differences between normal and degenerated human CEPs. **A,** When cleaned of remaining disc tissue, normal CEP was white, and appeared as normal hyaline cartilage. **B,** Degenerated CEP showed significant fibrosis and calcification. **C,** H&E staining of normal CEP revealed round cells with extensive deposition of extracellular matrix (arrow). **D,** Cells in degenerated CEP had multiple morphologies: round, polygonal and even spindle-like, and was invaded by blood vessels (arrow). Immunohistochemical staining and Western blotting analysis of ET-1 in degenerated CEPs. A relatively high intensity of staining was apparent in sections of degenerated CEPs. Brown staining was localized mainly in the cytoplasm (**F2**, arrow) and normal CEPs showed only few ET-1 stained cells (**E2**, arrow), **F1** and **E1** were negative controls, respectively. Protein samples at random were harvested from 2 normal and 2 degenerated fresh human CEPs. **G,** Western blotting was performed with an ET-1 antibody having a molecular weight of 24 kD. The control was GAPDH, with a molecular weight of 37 kD. The protein bands in lanes 3 and 4 from the patients with degenerated CEPs had significantly higher density than lanes 1 and 2. **H,** Relative expression of proteins, as shown by the ratio of ET-1 to GAPDH band densities. Assays were performed in triplicate and values are the means and standard deviations. *P<0.01 vs #1 (normal CEP), and ^#^P<0.01 vs #2 (normal CEP).

With H&E staining, normal CEP appeared as hyaline cartilage (Fig. 1A) after being cleared of both annulus fibrosus and nucleus pulposus. The extracellular matrix of CEP was homogeneous, with round-shaped CECs organized into obvious layers (Fig. 1C). Degenerated CEP had obvious fibrosis, fragmentation, ossification and occasional lysis of cell nuclei (Fig. 1B), accompanied by vascular invasion (Fig. 1D).

Immunohistochemical staining showed that most ET-1 immunoreactivity was present in degenerated CEPs; most cells showed positive reactions in the cytoplasm (Fig. 1F2). Normal CEPs contained few cells with positive ET-1 staining (Fig. 1E2).

Western blotting for ET-1 protein, assayed in normal CEPs (n = 2) and degenerated CEPs (n = 2), was present as a protein band with a molecular mass of 24 kDa (Fig. 1G and 1H). ET-1-specific protein was detected in all degenerated CEP samples; normal CEPs had a low ET-1 content.

### Morphology and Characterization of CECs *in vitro*

**Figure 2:**
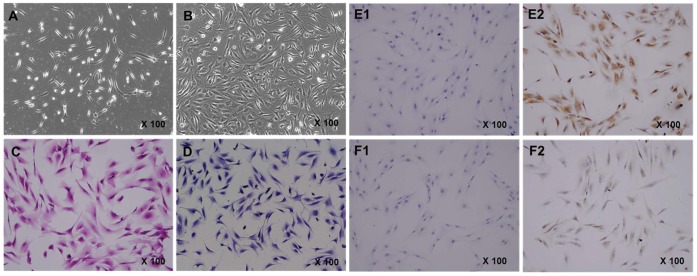
Primary culture of human cartilaginous endplate cells isolated from human degenerated CEP by type II collagenase digestion. Cells were obtained from five patients. **A,** After 7 days, cell colony-forming (CFU) had formed. **B,** After 14 days, the tissue culture flask was covered with polygonally shaped cells. The identification of human cartilaginous endplate cells. Morphological appearance and biological characteristics were determined by **C,** H&E staining and **D,** toluidine blue staining. With the positive expression of **E2,** collagen II and **F2,** aggrecan shown by immunocytochemical staining, **E1 and F1,** corresponding negative controls.

The primary culture cells were adherent at 12–24 h, and began to proliferate within 48 h. The cultured CECs formed colonies after approximately 7 days (Fig. 2A). When the primary cultures reached 90% confluence at about 14 days (Fig. 2B), the CECs exhibited various morphologies, which ranged from spindle-shaped to polygonal-shaped.

H&E staining showed that CECs had a mostly polygonal appearance, with a round or elliptical nucleus (Fig. 2C). The cytoplasm appeared blue with toluidine blue staining (Fig. 2D). Immunohistochemical staining of type II collagen (Fig. 2E2) and aggrecan (Fig. 2F2) was positive in the cytoplasm of CECs; no immunoreactivity was observed in the negative control groups (Fig. 2E1 and 2F1, respectively). Therefore, the phenotypes and biological characteristics of CECs were similar to those of articular chondrocytes.

### Immunofluorescence Staining of CECs for ET-1

**Figure 3:**
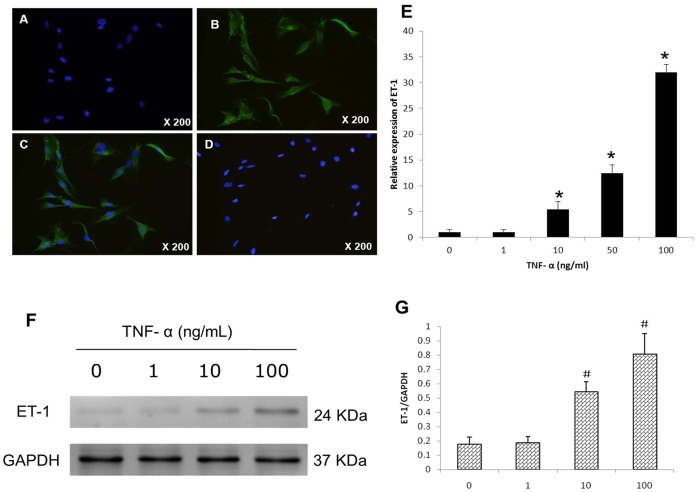
Immunofluorescence staining of CECs from degenerated cartilage endplate demonstrated intense ET-1 staining. **C,** Cytoplasmic staining by the merged image of **A**+**B**, with almost all cells showing green fluorescence of granular secretions. **D,** Controls showed only blue cell nuclei stained with DAPI. ET-1 mRNA and protein expression by CECs after TNF-α stimulation. Confluent CECs in six-well plates were synchronized in serum-free medium for 12 hours and then transferred in fresh serum-free DMEM. **E,** For ET-1 mRNA expression, TNF-α (0, 1, 10, 50,100 ng/ml) was added to cultures for 24 hours. Total RNA was extracted and reverse-transcribed for by real time PCR. The data was analyzed from three independent experiments. *P<0.01vs control. **F**, Expression of ET-1 protein levels correlated with mRNA, also in a dose-dependent manner. Cells were incubated with TNF-α (0, 1, 10,100 ng/ml) for 36 hours. Extracted proteins were analyzed by Western blotting, with GAPDH used as a loading control. **G,** Relative expression of ET-1 proteins compared with GAPDH. Statistical data were expressed as mean ± SD from three different samples. ^#^P<0.01 vs corresponding control without stimuli.

The presence of ET-1 in human degenerated CECs was evaluated by cell immunofluorescence staining. All CECs exhibited an intense positive reaction when incubated with the anti-ET-1-polyclonal antibody, with green fluorescence distributed primarily in the cytoplasm (Fig. 3C). No immunoreactivity was seen in control groups (Fig. 3D).

### Real-time PCR Analysis of ET-1 mRNA Expression

To establish ET-1 production by human CECs in response to TNF-α treatment, ET-1 mRNA expression was assessed by real-time PCR. Exposing CECs to TNF-α (0–100 ng/ml) for 24 h induced a concentration-dependent increase in ET-1 expression (Fig. 3E). At a TNF-α concentration of 10 ng/ml, there was a significant (5.44-fold) increase of ET-1 expression compared to that induced by the culture medium. The effect of TNF-α was less effective at a concentration of 1 ng/ml.

### Effect of TNF-α on Synthesis of ET-1 Protein by CECs

Western blot analysis of the effect of TNF-α (0–100 ng/ml) on ET-1 protein synthesis is shown in Fig. 3F and Fig. 3G. After TNF-α treatment for 36 h, the ET-1 protein concentrations significantly increased in a dose-dependent manner, which was in accord with the real-time PCR results. Therefore, the pro-inflammatory effects of TNF-α resulted in ET-1 increases not only at the mRNA level, but also in protein production.

### Effects of ET-1 on MMP-1, MMP-13 and TIMP-1 Release

**Figure 4:**
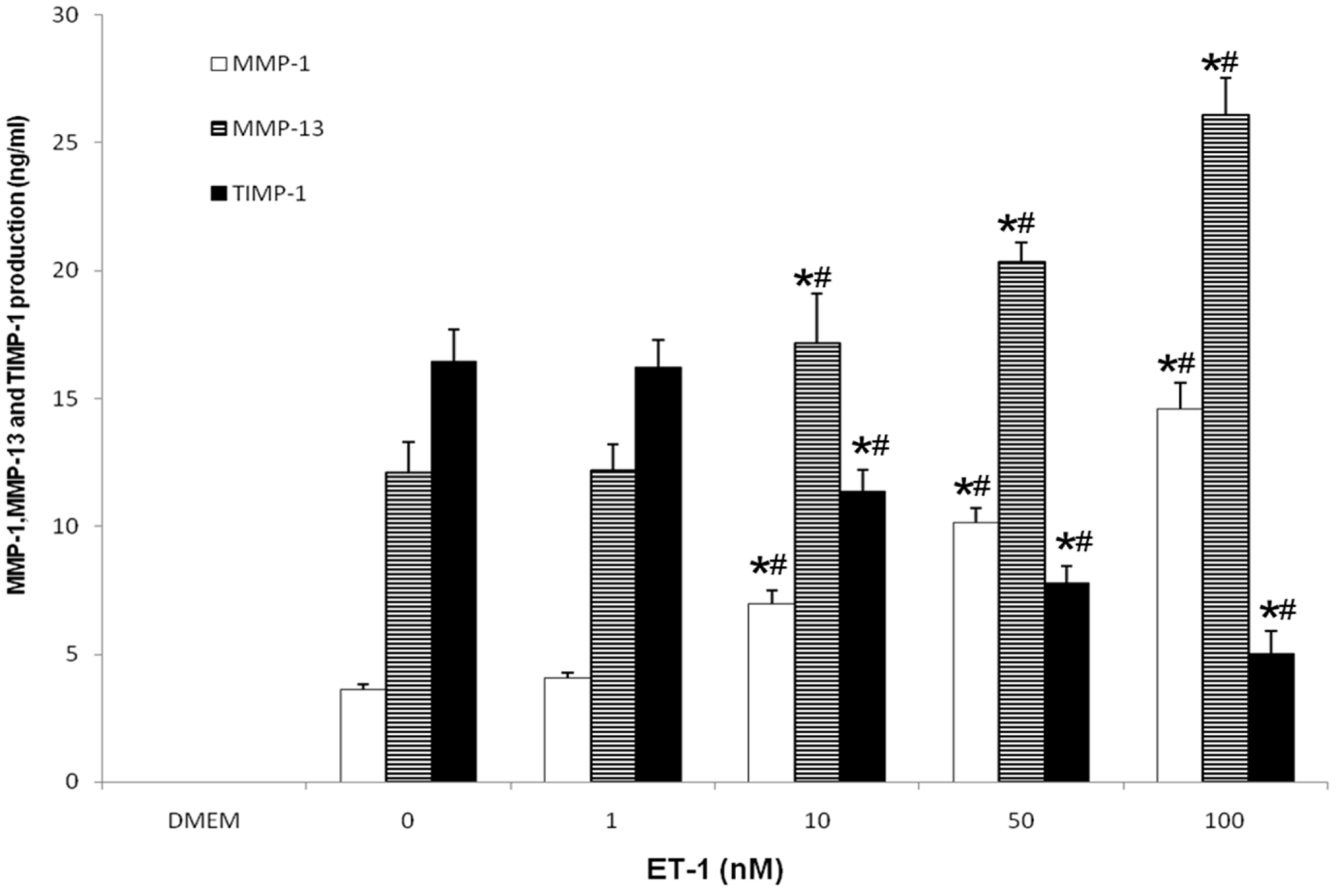
ET-1 upregulated MMP-1 and MMP-13 expression and downregulated TIMP-1 release. Confluent CECs in six-well plates were cultured in serum-free medium for 12 hours and then treated with ET-1 (0–100 nM) for 36 hours. ET-1 increased the release of MMP-1 and MMP-13, but decreased the production of TIMP-1 in a dose-dependent pattern as shown by ELISA assay. The results represent the mean ± SD in triplicate. *P<0.01 vs DMEM group, ^#^P<0.01 vs corresponding control without stimuli.

To assess the importance of ET-1 in the induction of MMP-1 and MMP-13 production and TIMP-1 activity, CECs were treated with ET-1 (0–100 nM) for 36 h. MMP-1 and MMP-13 secretion increased in a dose-dependent pattern, whereas TIMP-1 correspondingly decreased as analyzed by ELISA (Fig. 4). It was shown that certain amounts of both MMP-1 and MMP-13 were still released in the absence of ET-1; however, the production of TIMP-1 was higher than the MMPs. Following stimulation with added ET-1, the syntheses of MMP-1 and MMP-13 were both far greater than that of TIMP-1.

### Effects of ET-1 on NO and NOS Production

**Figure 5:**
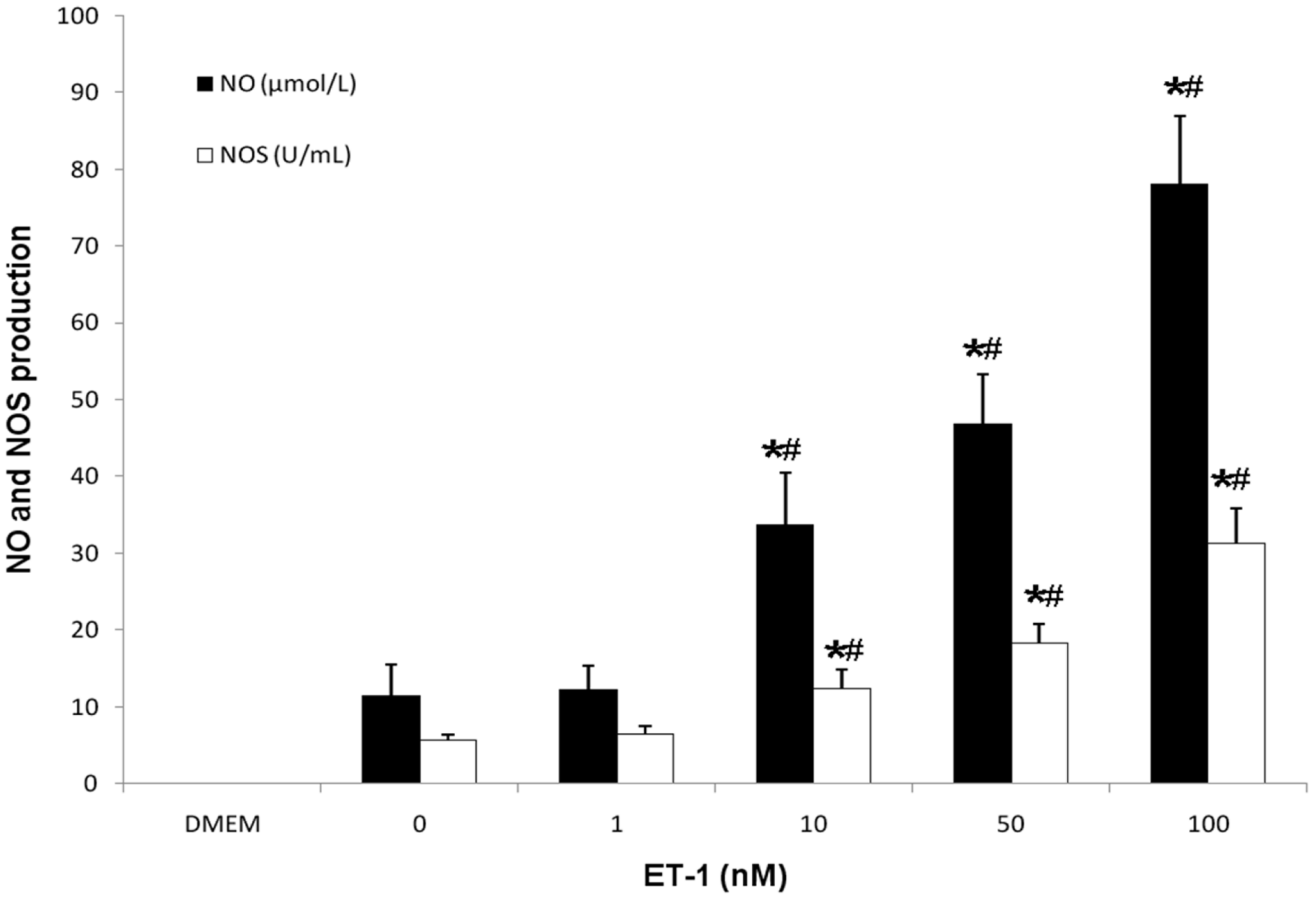
ET-1 stimulated NO and NOS release. After synchronization in serum-free medium for 12 hours, CECs were treated with ET-1 (0, 1, 10, 50, 100 nM) for 36 hours. ET-1 increased NO release and NOS activity in a dose-dependent manner. Statistical data were expressed as mean ± SD from three different experiments. *P<0.01 vs DMEM control, ^#^P<0.01 vs corresponding control without stimuli.

A 36 h incubation of CECs with ET-1 (0–100 nM) resulted in a statistically significant dose-dependent increase of both NO release and NOS production, as shown in Fig. 5. The differences in both NO and NOS were significant for an ET-1 concentration of 10 nM ET-1 compared with either 1 nM (p<0.05) or with the culture medium control (p<0.05). The results demonstrated that NO release increased along with NOS activity.

### ET-1 did not Induce Apoptosis

**Figure 6:**
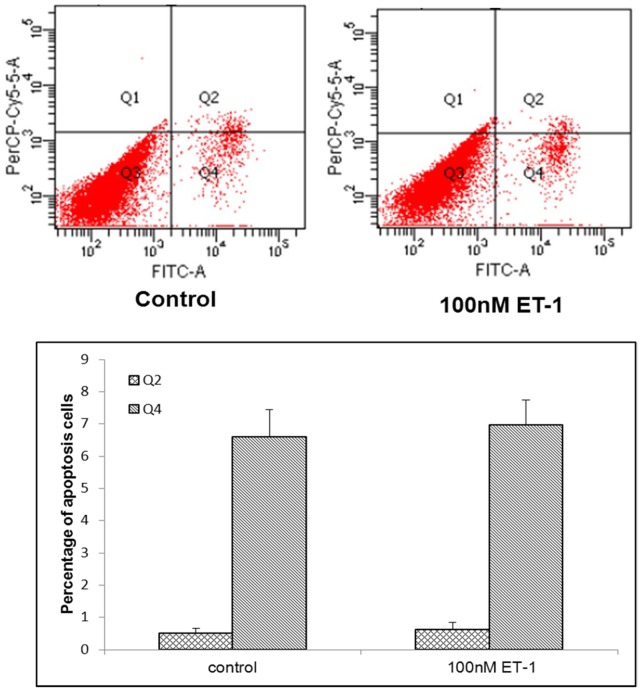
ET-1 did not induce apoptosis. Confluent CECs were incubated with ET-1 (0–100 nM) plus 2% FCS+DMEM medium for 72 hours. The apoptosis rates of CECs were examined by an annexin V-FITC/PI method. No significant differences of cell apoptosis, compared with unstimulated controls, were detected by flow cytometric analysis (Only data from controls and cultures treated with 100 nM ET-1 were shown). The results were analyzed by mean ± SD of three experiments.

The Treatment of CECs by ET-1 (0–100 nM) for 72 h did not have an effect on apoptosis as measured by the Annexin V/PI assay (Fig. 6). In the presence of 100 nM ET-1, there were similar percentages of positively stained CECs compared with the control groups without ET-1 treatment. In the early stage of apoptosis, the Q4 proportions were 6.97% ±1.35% and 6.6% ±1.44%, respectively; and at the later stage of apoptosis, the Q2 proportions were 0.63% ±0.37% and 0.5% ±0.29%. Neither difference was significant (p>0.05).

## Discussion

The nutrient supply of IVDs is primarily provided by endplate diffusion [18]. Therefore, endplate degeneration is directly related to disc degeneration [19]–[21]. However, the exact mechanism of endplate degeneration remains to be elucidated. Several studies have pointed out that inflammatory responses, namely a variety of pro-inflammatory or inflammatory cytokines, may be involved in IVD degeneration [22], [23].

ET-1 was first reported in cultures of porcine aortic endothelial cells and since then has been found in many cell types, including mesangial, granulosa and bone cells [24]–[26]. In addition, it has been shown that cultured rat articular chondrocytes synthesize and release substantial amounts of ET-1 [27]. Furthermore, Roy-Beaudry *et al*. reported the expression and synthesis of ET-1 in human joint cartilage and synovial membrane tissues, where ET-1 was associated with degenerative disease [16]. Concerning the similarity between joint cartilage and endplate cartilage, we postulated that ET-1 may be implicated with CEP degeneration in IVDs. Encouragingly, this study supports our hypothesis that ET-1 has a role in the pathogenesis of endplate degeneration.

To our knowledge, this is the first report to demonstrate the expression and synthesis of ET-1 in human CEP. We found that the expression of ET-1 in degenerated endplate tissues was far higher than in non-degenerate samples, according to IHC staining and western blotting analysis. The results suggest that degeneration of intervertebral endplates may be attributable to abnormally high ET-1 expression. Possible cellular and molecular mechanisms of ET-1 action were investigated in isolated CECs from degenerated CEP. In degenerated discs, there may be pro-inflammatory cytokines that trigger over-expression of other molecules, resulting in matrix degradation or promoting catabolic processes. Kang *et al*. reported that exogenous interleukin-1 (IL-1) induced the production of NO, interleukin-6 (IL-6), prostaglandin E2 (PGE_2_) in IVDs, which inhibited proteoglycan synthesis [28]. In many other inflammatory injury systems, a transient increase in TNF-α has been found to act as an inducer of other more long lasting cytokines. In addition, TNF-α is known to be a key mediator of pain and inflammation. Significantly increased levels of TNF-α occur in herniated and degenerate IVD tissue, and are associated with radiculopathy following herniation [29]. In this context, we chose the pro-inflammatory cytokine TNF-α, as an inflammatory stimulus in cultured CECs. Subsequent increased ET-1 mRNA levels were detected in the CECs after 24 h of exposure. Importantly, this was correlated with an increased ET-1 levels in the culture medium, as determined by western blotting, after 36 h of incubation. This is in line with previous reports that ET-1 production is increased and regulated by growth factors and cytokines such as IL-1ß, TNF-α, LPS, TGF- ß1, EGF, and IGF-I in rat articular chondrocytes [30]. The biologic effect of one cytokine is often modified or augmented by another, but the exact mechanisms are still unclear.

It is generally accepted that progressive tissue destruction in IVD degeneration results from over-expression of various proteolytic enzymes and other catabolic agents [31], [32]. Action of MMPs is a crucial event in IVD degeneration by initiating matrix catabolism and then IVD degeneration [33], [34]. In our study, MMP-1 and MMP-13 levels in CECs increased significantly in response to ET-1 in a dose-dependent manner, whereas TIMP-1 correspondingly decreased. MMP-1 and MMP-13 are collagenolytic enzymes that play an important role in the alteration of collagen in degenerated discs; especially MMP-13, which cleaves extracellular matrix proteins including type II collagen [35], [36]. The results demonstrate that the effect of ET-1 was greater on MMP-13 production than on MMP-1. Generally, MMP enzymes are maintained in an inactive form by tissue inhibitors. Our results show that ET-1 significantly increased MMP-1 and MMP-13 release in CECs, affecting the balance of MMP/TIMP and presumably assisting in the degradation of the extracellular matrix. In addition, NO has been shown to mediate changes in proteoglycan synthesis in human IVDs as well as pain-related behavior in a rat model of radiculopathy [8], [37], so excess NO is harmful to cells. Our results showed that NO levels significantly increased in a dose-dependent manner with ET-1 treatment. Therefore, the action of ET-1 seems to depend on increases in both MMP and NO production. In the study, CECs derived from human degenerated disc samples, may be subject to TNF-α induction *in vivo*, and thus may release a certain amount of ET-1 in the absence of any exogenous stimulation. During the process of CEP degeneration seen in our tissue samples, blood vessels from bone marrow of the vertebral body invaded the degenerated endplate tissue (Fig. 1D), which is also known to secrete ET-1. We suggest that ET-1 may not only be secreted by CECs but also be released by invasive blood vessels. They may act synergistically in CEP degradation.

Wang *et al.* reported that the vertebral endplate degeneration was related to endplate cell apoptosis [38]. We found that ET-1 did not act on IVD degeneration through induction of apoptosis. Others have reported similar results of ET-1 on articular chondrocytes without apoptosis [13].

In conclusion, our results showed associations between ET-1 protein levels and a number of changes in cultured CECs from degenerated endplates suggesting that ET-1 functions as a mediator in the etiology of disc degeneration. Future biologic treatments including inhibitors or blockers of ET-1 or various intracellular signaling molecules may potentially help replenish the matrix content by decreasing catabolic processes.
